# LINC01303 functions as a competing endogenous RNA to regulate EZH2 expression by sponging miR‐101‐3p in gastric cancer

**DOI:** 10.1111/jcmm.14593

**Published:** 2019-09-09

**Authors:** Chen Cao, Ying Xu, Ke Du, Chenyang Mi, Chuanhua Yang, Lili Xiang, Yan Xie, Wenneng Liu

**Affiliations:** ^1^ West China School of Public Health and West China Fourth Hospital Sichuan University Chengdu China; ^2^ BioBank The First Affiliated Hospital of Xi'an Jiaotong University Shaanxi China

**Keywords:** EZH2, gastric cancer, LINC01303, metastasis, miR‐101‐3p, proliferation

## Abstract

Long non‐coding RNA (lncRNA) is one of the important regulators of many malignancies. However, the biological function and clinical significance of a large number of lncRNAs in gastric cancer remain unclear. Therefore, we analysed the TCGA data to find that LINC01303 is significantly up‐regulated in gastric cancer tissues. However, the biological function of LINC01303 in GC remains unknown. In our study, we found that the expression of LINC01303 was significantly higher in GC tissues than in adjacent tissues by real‐time quantitative PCR. We can significantly inhibit the malignant proliferation, migration and invasion of GC cells by silencing LINC01303 expression. In addition, LINC01303 knockdown can also inhibit GC growth in vivo. After the bioinformatics analysis, we found that LINC01303 can be used as a miR‐101‐3p sponge to competitively adsorb miR‐101‐3p with EZH2. Therefore, our results indicate that LINC01303 promotes the expression of EZH2 by inhibiting miR‐101‐3p activity and promotes GC progression. In summary, in this study, we demonstrated for the first time that the LINC01303/miR‐101‐3p/EZH2 axis promotes GC progression.

## 1 INTRODUCTION

Gastric cancer (GC) is one of the major malignancies with high mortality worldwide.^1^, ^2^ Despite significant advances in the study of the pathogenesis of GC, treatments for GC remain limited.^3^, ^4^ In addition, recent studies have shown that the pathogenesis behind GC remains largely unknown.^5^, ^6^ Therefore, clinical diagnosis and treatment require new predictive biomarkers and their function in the GC to reveal GC benchmarks.

Long non‐coding RNA (lncRNA) is a recently discovered member of the non‐coding RNA family.^7^ Recently, there is increasing evidence that non‐coding RNAs (ncRNAs) play a key role in human cancer biology.^7^, ^8^ These ncRNA molecules over 200 nucleotides in length are named long non‐coding RNAs (lncRNAs) that regulate mRNA translation, transcriptional processes and cell development, differentiation, proliferation and apoptosis.^9^ There is increasing evidence that lncRNA can be used as a biomarker for the diagnosis and prognosis of various cancers, such as colorectal cancer,^10^, ^11^, ^12^ breast cancer,^11^, ^12^ breast cancer,^13^ liver cancer^14^ and gastric cancer.^15^, ^16^ In addition, in the study of liver metastases from colorectal cancer, it was found that lncRNA UICLM inhibits the expression of miR‐150‐5p by competitive endogenous RNA, thereby promoting liver metastasis of colorectal cancer.^17^, ^18^ Long non‐coding RNA can not only regulate gene expression as a competitive endogenous RNA, but also regulate epigenetics by binding to related proteins. For example, lncRNA HOTAIR promotes metastasis of gastric cancer by binding to the epigenetic transcriptional regulator polycomb inhibitor complex 2 (PRC2).^19^, ^20^

In this study, we first found that LINC01303 was significantly up‐regulated in GC tissue and was associated with poor prognosis. Loss and functional gain assays showed that LINC01303 affects GC cell proliferation, migration and invasion by acting as a miR‐101‐3p ceRNA, thereby preventing miR‐101‐3p from binding to the target protein EZH2. Overall, the results indicate that LINC01303 is an important oncogenic regulator in the development of tumours in GC and may be a potential target for clinical diagnosis and treatment of GC.

## 2 MATERIAL AND METHODS

### 2.1 Human samples

Human GC tissue (n = 30) and matched adjacent non‐tumour tissue (n = 30) were obtained from surgical patients in the Fourth Hospital of Western China. Two pathologists evaluated the histological evaluation of each sample in a double‐blind manner. All GC patients have signed written informed consent, and all procedures are based on the Clinical Research Ethics Committee of the Fourth Hospital of West China.

### 2.2 Cell lines and treatment

Human GC cell lines BGC823, SGC 7901, MKN‐45, AGC and immortalized human gastric epithelial cell line GES‐1 were obtained from Cell bank of Chinese Academy of Sciences (Shanghai, China), and no evidence of mycoplasma contamination was detected by using PCR‐based assay test. Cells were cultured in DMEM medium (Gibco) supplemented with 10% FBS (Gibco) and 1% penicillin/streptomycin in 5% CO_2_ at 37°C.

### 2.3 Cell proliferation assay

For the Cell Counting Kit‐8 (CCK‐8) assay, cells were seeded into 96 well plates of 5 × 10^3^ cells/well. CCK‐8 (Dojindo) was added to each well and incubated at 37°C for 2 hours. For colony formation assay, 1 × 10^3^ cells were inoculated without holes in a 6‐well plate, and after 14 days of culture, crystal violet staining was counted.

### 2.4 Subcutaneous xenograft nude mouse studies

Six‐week BALB/C male nude mice were used for in vivo studies. 5 × 10^6^ cells in 0.2 mL PBS were injected subcutaneously into the dorsal side (n = 3/group). A nude mouse tumour metastasis model was prepared, and a 5 × 10^6^ cell passage was injected into the nude mouse tail vein to the nude mice. Tumour weight was measured after the mice were killed. Tumours were collected and prepared for histological examination.

### 2.5 Statistical analysis

We performed statistical analysis using Prism 5 and SPSS 18.0 software. Differences between the two groups were assessed using Student's *t* test. A *P* value < .05 was considered to represent a significant difference. The overall survival curve was estimated by the Kaplan‐Meier method and the Cox proportional hazard model. All values are expressed as mean ± SD unless otherwise stated.

## 3 RESULTS

### 3.1 LINC01303 is up‐regulated in GC and associated with poor prognosis

**Figure 1:**
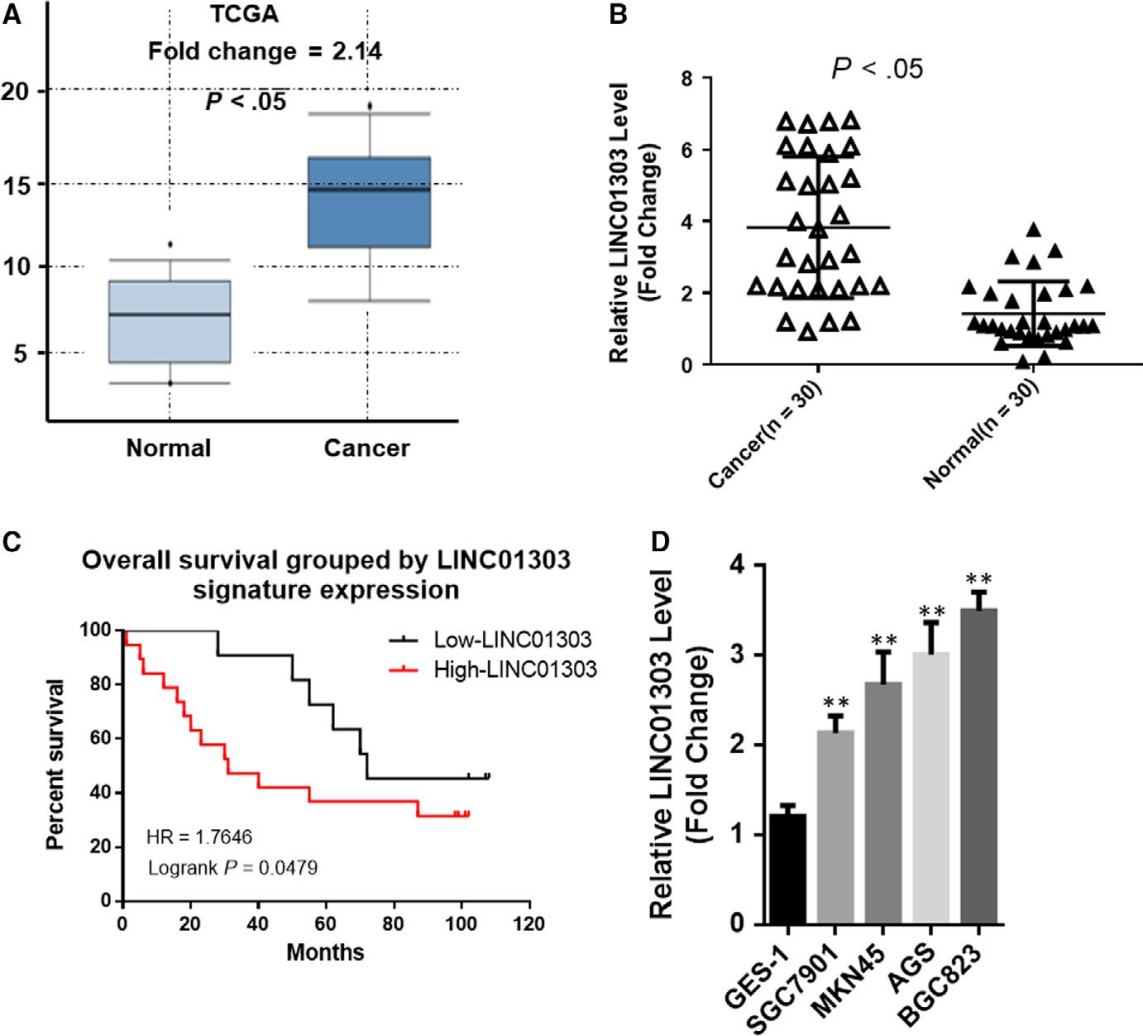
LINC01303 is up‐regulated in GC and associated with poor prognosis. A, LINC01303 levels were increased in GC tissues compared with paired adjacent non‐tumour tissues as determined by The Cancer Genome Atlas. B, LINC01303 levels were increased in GC tissues compared with paired adjacent non‐tumour tissues as determined by real‐time PCR. Data are mean ± SD. n = 30, *P < .05. C, Based on the expression level of LINC01303, the Kaplan‐Meier curve depicts the overall survival curve of 30 patients with gastric cancer. D, Real‐time quantitative PCR was used to analyse the expression of LINC01303 in normal gastric epithelial cell line (GES‐1) and gastric cancer cells. Error bars indicate mean ± SE of the mean. **P < .01

To investigate the correlation between LINC01303 and human GC, we found that the mRNA expression of LINC01303 in GC tissues was up‐regulated compared to normal gastric tissue by searching public data from the Cancer Genome Atlas (TCGA) database (Figure 1A). We also analysed the mRNA expression levels of LINC01303 in 30 pairs of GC tissues and adjacent non‐tumour tissues by real‐time PCR assay. The results showed that the expression of LINC01303 in GC tissues was significantly higher than that in non‐tumour tissues (Figure 1B, *P* < .05). To assess the clinical significance of LINC01303 expression in GC, we assessed the association between LINC01303 levels and clinicopathological features. As shown in Table 1, high expression of LINC01303 was associated with patient age (*P* = .018), larger tumour size (*P* = .001), depth of invasion (*P* = .010) and AJCC stage (*P* = .001). We analysed the relationship between the expression level of LINC01303 and the prognosis of patients with GC by Kaplan‐Meier survival. Kaplan‐Meier survival analysis showed that patients with higher levels of LINC01303 had shorter overall survival than patients with lower levels of LINC01303 (Figure 1C). At the same time, LINC01303 was significantly overexpressed in GC cell lines compared to human normal gastric epithelial cells (GES‐1) (Figure 1D). These data indicate that LINC01303 is involved in the pathogenesis of gastric cancer (GC).

**Table 1:** LINC01303 levels and clinicopathological features in 30 GC patients

Characteristics	Total	LINC01303		*P*
		Low	High	
Gender				
Female	13	6	7	1.000
Male	17	8	9	
Age (y)				
>60	18	5	13	.018
≤60	12	2	10	
Tumour size (cm)				
<10	14	12	2	.001
≥10	16	3	13	
Invasion depth				
Without Infiltration into Serous layer	20	9	11	.010
Infiltration into Serous layer	10	1	9	
AJCC stage				
I\II	12	8	4	.001
III\IV	18	2	16	

### 3.2 LINC01303 promoted cell proliferation of GC in vitro and in vivo

**Figure 2:**
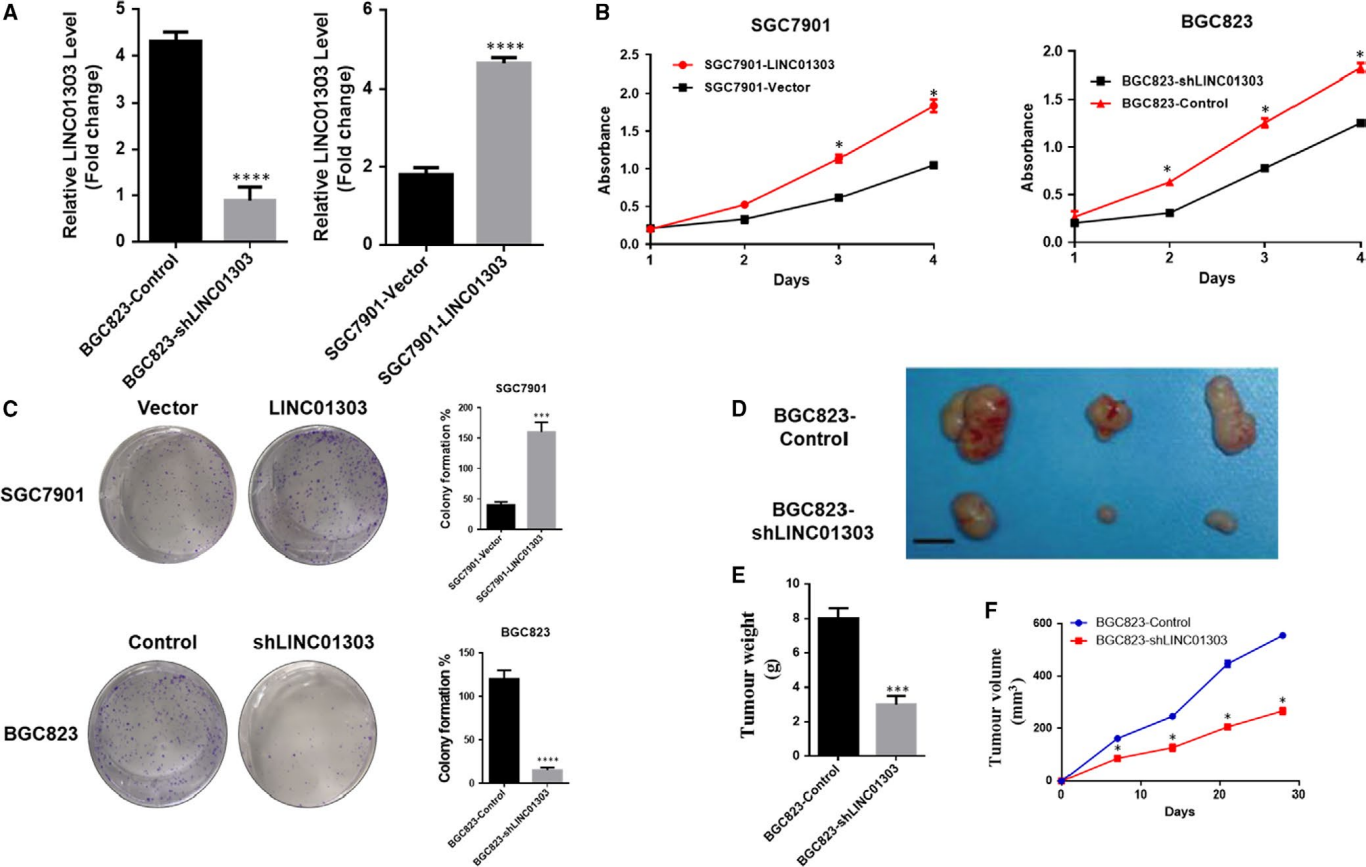
LINC01303 promoted cell proliferation of GC in vitro and in vivo. A, Real‐time quantitative PCR analysis of LINC01303 expression in with the lentivirus SGC 7901‐LINC01303 and with BGC 823‐shLINC01303 for further investigation. B, The CCK8 assay was used to determine the viability of BGC823‐shLINC01303 transfected or SGC7901‐LINC01303 transfected gastric cancer cells. C, Colony formation assays were performed to determine the proliferation of BGC823‐shLINC01303‐transfected or SGC7901‐LINC01303‐transfected gastric cancer cells. D, Stable shLINC01303 knockdown BGC823 cells were used for in vivo assays. E, Represents tumour weight from both groups (n = 3). F, Tumours from two groups of nude mice were shown and measured and showed tumour growth curves after injection of BGC823 cells. Tumour volume was calculated every 4 d. The data represent the mean ± SD from three independent experiments. *P < .05, **P < .01, ***P < .001

To elucidate the effect of LINC01303 on GC cell proliferation, we overexpressed or knocked out LINC01303 to construct SGC7901‐LINC01303 cells stably transfected with BGC823‐shLINC01303 cells for further study (Figure 2A). In order to analyse the effect of knockout or overexpression on the proliferation of gastric cancer cells, we showed that the overexpression of LINC01303 significantly increased the cell proliferation of GC cells by CCK8 and colony formation experiments, while the down‐regulation of LINC01303 significantly inhibited the proliferation of GC cells (Figure 2B,C). At the same time, in order to prove that LINC01303 can also affect the proliferation of gastric cancer in vivo, we further confirmed that LINC01303 significantly promoted the tumorigenicity of GC by subcutaneous xenograft nude mouse model (Figure 2D‐F). Taken together, these data indicate that LINC01303 contributes to the proliferation of GC cells.

### 3.3 LINC01303 accelerates GC cell migration and invasion in vitro and in vivo

**Figure 3:**
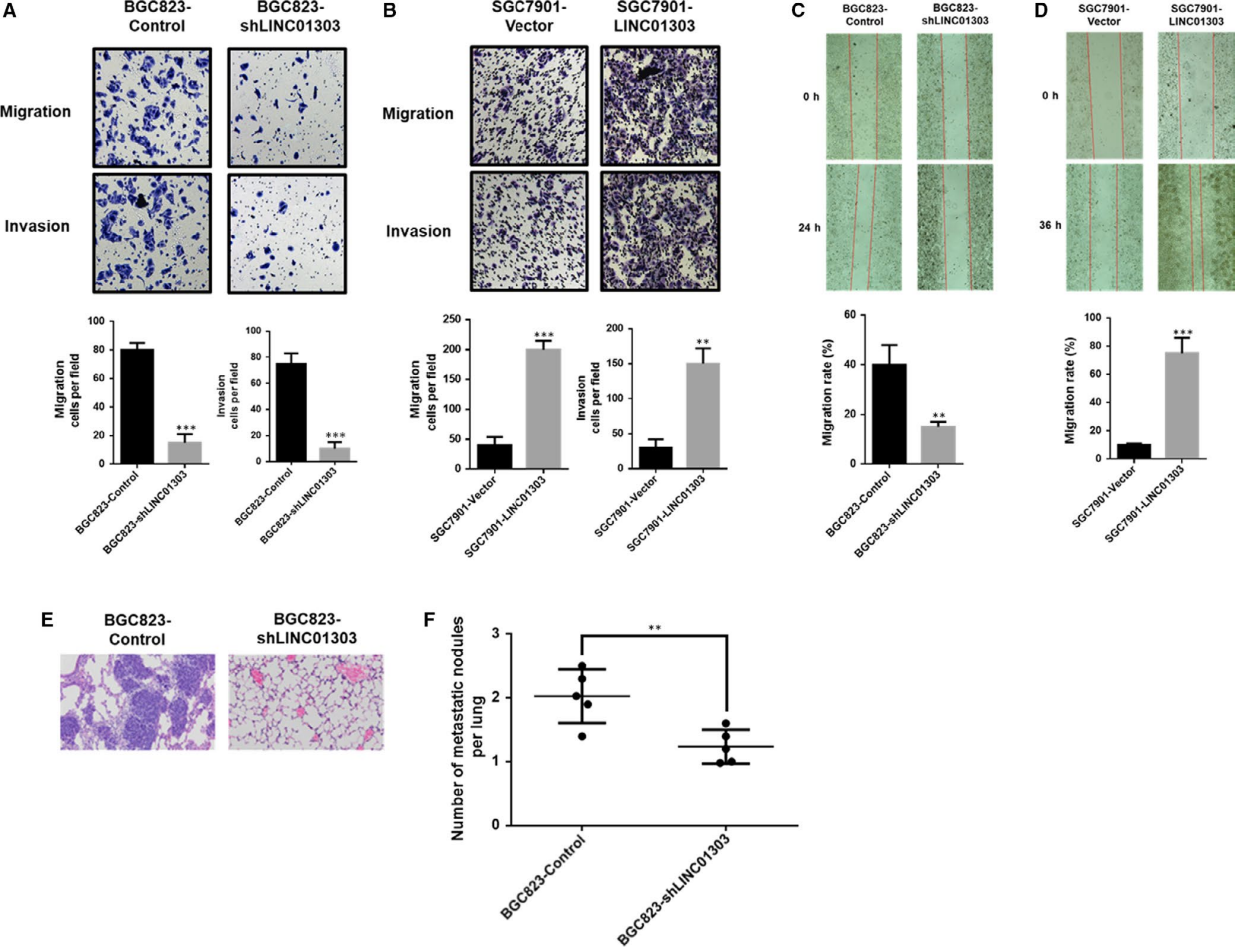
LINC01303 accelerates GC cell migration and invasion in vitro and in vivo*.* A, Matrigel migration and invasion assay of GC cells lenti‐shLINC01303; B, Matrigel migration and invasion assay of GC cells lenti‐LINC01303; C, wound healing assay of GC cells lenti‐shLINC01303; D, wound healing assay of GC cells lenti‐LINC01303. E, F, Representative HE stained sections of lung metastasis nude mice bearing BGC823 control cells or BGC823 shLINC01303 cells (n = 5). The data represent the mean ± SD from three independent experiments. *P < .05, **P < .01, ***P < .001

To clarify whether LINC01303 is capable of modulating GC cell migration and invasion. We performed wound healing and transwell experiments. The results showed that overexpression of LINC01303 significantly promoted the migration and invasion of GC cells (Figure 3B). Conversely, knockdown of LINC01303 significantly inhibited the migration and invasion of GC cells (Figure 3A). Similarly, wound healing experiments further demonstrated that a more efficient wound closure rate was observed in cells overexpressing LINC01303, while a lower wound closure rate was detected in LINC01303 knockdown cells (Figure 3C,D). To demonstrate, in addition, the lung metastasis nude mouse model further confirmed that LINC01303 significantly promoted GC metastasis in vivo. (Figure 3E,F), indicating that LINC01303 promotes GC progression by modulating cell migration and invasion in vitro and in vivo.

### 3.4 LINC01303/miR‐101‐3p/EZH2 axis regulates GC progression

**Figure 4:**
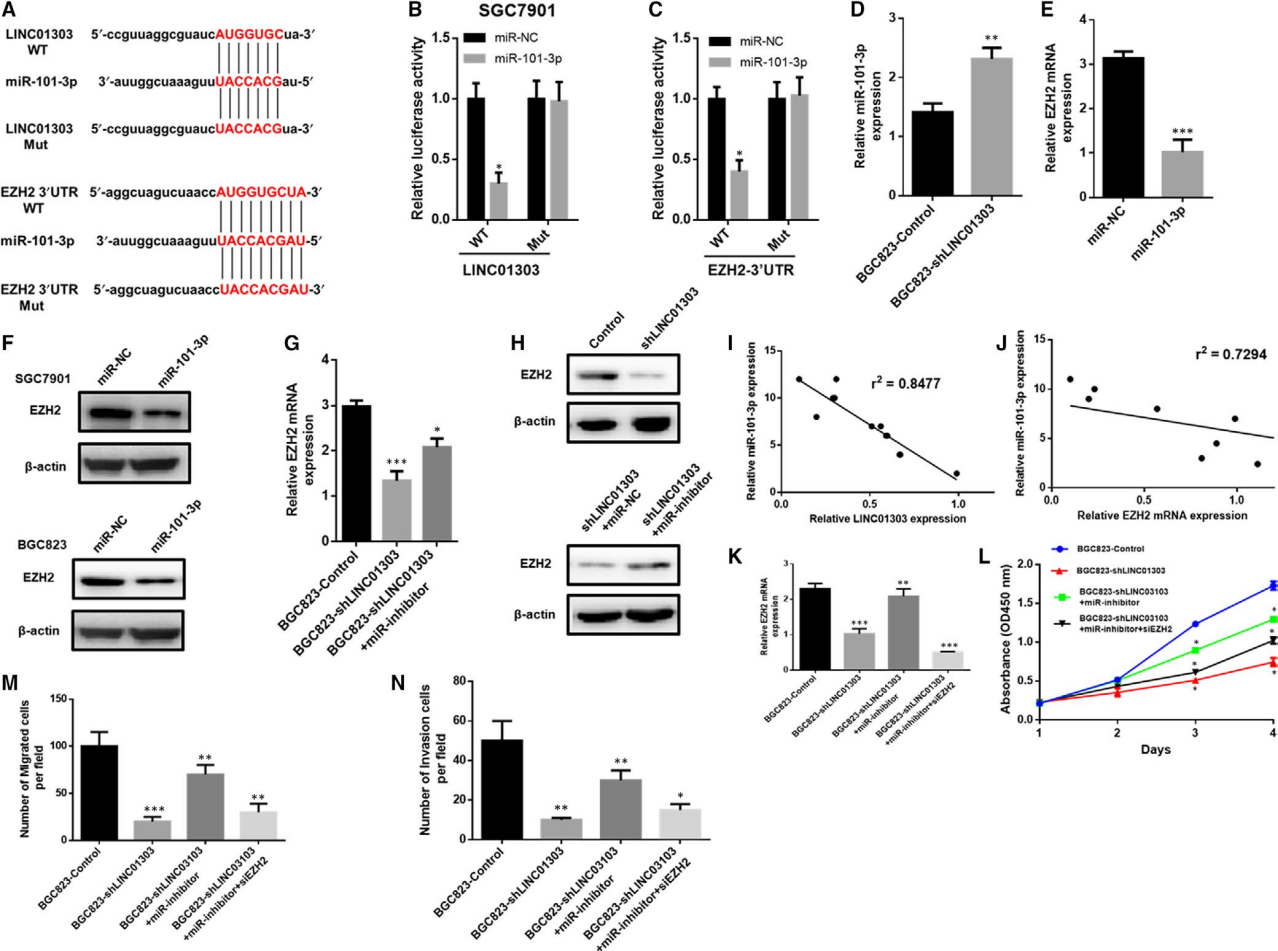
LINC01303/miR‐101‐3p/EZH2 signalling regulates GC progression. A, miR‐101‐3p‐binding sequences in LINC01303 or the 3′‐UTR of EZH2 and mutant sites in LINC01303 or the 3′‐UTR of EZH2. B, C, Luciferase reporter assay showed that the luciferase activity of either LINC01303‐WT or EZH2 3′‐UTR‐WT was inhibited by miR‐101‐3p mimics. D, miR‐101‐3p expression was increased in BGC823 cells after LINC01303 silencing. E, F, EZH2 expression was reduced after transfection with miR‐101‐3p mimics. G, H, LINC01303 regulated EZH2 expression in BGC823 cells through inhibitor miR‐101‐3p. I, The expression of LINC01303 was negatively correlated with miR‐101‐3p expression in GC tissues. J, EZH2 expression was reversely correlated with miR‐101‐3p level in GC tissues. K, Expression of EZH2 was measured in BGC823 cells after transfection with described vectors. L, CCK8 assay was used to analyse cell proliferation. M, N, Transwells assay was exploited to measure migration and invasion in BGC823 cells. The data represent the mean ± SD from three independent experiments. *P < .05. **P < .01, ***P < .001

Then, we found that LINC01303 may act as a sponge to adsorb miR‐101‐3p. Further predictions suggest that miR‐101‐3p may target the 3'UTR of EZH2. Next, we designed the luciferase reporter gene experiment, as shown in Figure 4A. The results showed that the miR‐101‐3p simulation significantly inhibited the reporter gene activation ability of LINC01303‐WT and EZH2‐WT (Figure 4B,4). However, the miR‐101‐3p mimic has no inhibitory ability to the mutated luciferase reporter gene of the binding site (Figure 4B,4). These results suggest that miR‐101‐3p may interact with LINC01303 and EZH2. RT‐PCR results also showed that LINC01303 knockdown increased the expression level of miR‐101‐3p (Figure 4D), while miR‐101‐3p mimic significantly inhibited the expression level of EZH2. (Figure 4E). Western blot confirmed that miR‐101‐3p inhibited EZH2 expression in SGC7901 and BGC823 cells (Figure 4F). At the same time, we have been suggested that LINC01303 may regulate the expression level of EZH2 by competitively adsorbing miR‐101‐3p. To clarify this hypothesis, we knockdown LINC01303 and found that EZH2 expression levels were reduced, while miR‐101‐3p expression levels were up‐regulated (Figure 4G,H). Importantly, we found for the first time that miR‐101‐3p expression levels were negatively correlated with LINC01303 or EZH2 expression levels in GC tissues (Figure 4I,J). In summary, our results demonstrate that LINC01303 regulates the miR‐101‐3p/EZH2 axis in GC. To further confirm the role of LINC01303 in regulating miR‐101‐3p/EZH2 axis in GC progression, we performed experiments using miR‐101‐3p inhibitor and EZH2 siRNA. To confirm transfection efficiency, EZH2 expression was tested in BGC823 cells transfected with the plasmid (Figure 4K). CCK8 and transwell assays were then performed to analyse proliferation, migration and invasion. To elucidate the role of the LINC01303/miR‐101‐3p/EZH2 axis in gastric cancer, we performed experiments using the miR‐101‐3p inhibitor and EZH2 siRNA. EZH2 expression was tested in transfected BGC823 cells (Figure 4K). We then found that LINC01303 silenced inhibition of proliferation (Figure 4L), migration and invasion (Figure 4M,N). The addition of the miR‐101‐3p inhibitor reversed the effect of sh‐LINC01303 (Figure 4L). In addition, the effect of the miR‐101‐3p suppressor was further reversed by knockdown of EZH2 (Figure 4M,N). In summary, the LINC01303/miR‐101‐3p/EZH2 axis plays a key role in regulating GC progression.

## 4 DISCUSSION

There is increasing evidence that long non‐coding RNAs have great potential applications in the diagnosis and treatment of GC.^9^, ^21^ In our study, we first demonstrated that the expression of LINC01303 in GC tissues was higher than that in adjacent tissues.

Overall, the mechanism of action of lncRNA is mainly to inhibit the expression of downstream target genes by inhibiting the expression of miRNAs.^7^, ^22^ Therefore, the downstream target gene of miRNA is one of the important components of the ceRNA network. For example, LINC01234 inhibits GC cell proliferation mainly through mucus adsorption of miR‐204‐5p, thereby affecting the expression of miR‐204‐5p downstream target genes.^20^, ^23^ There are also reports that the long non‐coding RNA NEAT1‐2 adsorbs miR‐106b‐5p through the sponge, thereby up‐regulating the expression of the ATAD2 gene in PTC to promote tumorigenesis and progression.^24^ LncRNA‐HOXA11‐AS adsorbs miR‐1297 through ceRNA mechanism to promote the proliferation and metastasis of GC cells.^25^ Therefore, in order to clarify the mechanism by which LINC01303 promotes the development of gastric cancer. We conducted a series of in vitro and in vivo experiments. Overexpression of LINC01303 significantly promoted cell proliferation as demonstrated by CCK‐8 assay and colony formation assays as well as subcutaneous xenograft models in nude mice.

In conclusion, our results indicate that LINC01303 can up‐regulate EZH2 expression as a miR‐101‐3p miRNA sponge, thereby promoting the malignant behaviour of GC cells. Our work suggests that the LINC01303/miR‐101‐3p/EZH2 axis may be a potential target for clinical diagnosis and treatment of GC.

## CONFLICT OF INTEREST

The authors have no conflicts of interest to disclose.

## AUTHOR CONTRIBUTIONS

WNL conceived of and directed the project. CC and YX designed and performed the experiments. XY conducted the data analysis and interpreted the results. CYM, CHY and LLX helped to design the study. CC and YX wrote and edited the manuscript.

## Data Availability

The data that support the findings of this study are available from the corresponding author upon reasonable request.
